# Bead Based Proteome Enrichment Enhances Features of the Protein Elution Plate (PEP) for Functional Proteomic Profiling

**DOI:** 10.3390/proteomes3040454

**Published:** 2015-12-08

**Authors:** Xing Wang, Michael Davies, Swapan Roy, Matthew Kuruc

**Affiliations:** 1 Array Bridge, Inc., St. Louis, MO 63108, USA; E-Mails: xing.wang@arraybridge.com (X.W.); michael.davies@arraybridge.com (M.D.); 2 Biotech Support Group LLC, Monmouth Junction, NJ 08852, USA; E-Mail: swapan@biotechsupportgroup.com

**Keywords:** functional proteomics, 2-D gel, PEP, biomarkers, protein enrichment, cancer

## Abstract

A novel functional proteomics technology called PEP(Protein Elution Plate) was developed to separate complex proteomes from natural sources and analyze protein functions systematically. The technology takes advantage of the powerful resolution of two-dimensional gel electrophoresis (2-D Gels). The modification of electrophoretic conditions in combination with a high-resolution protein elution plate supports the recovery of functionally active proteins. As 2DE(2-Dimensional Electrophoresis) resolution can be limited by protein load, we investigated the use of bead based enrichment technologies, called AlbuVoid™ and KinaSorb™ to determine their effect on the proteomic features which can be generated from the PEP platform. Using a variety of substrates and enzyme activity assays, we report on the benefits of combining bead based enrichment to improve the signal report and the features generated for Hexokinase, Protein Kinase, Protease, and Alkaline Phosphatase activities. As a result, the PEP technology allows systematic analysis of large enzyme families and can build a comprehensive picture of protein function from a complex proteome, providing biological insights that could otherwise not be observed if only protein abundances were analyzed.

## 1. Introduction

The field of proteomics endeavors to systematically identify, construct and finally contrast proteomes based on protein quantities or abundances of individual gene products. Ideally it strives to ascribe function to these same gene products. The role of protein function and how such function is altered in disease states is of foremost importance to our understanding of health, well-being and potential for therapeutic intervention. Defining the roles of functional proteins such as enzymes and the understanding of their interaction network in the cell and various tissues is of central importance in the elucidation of basic biological processes and important disease mechanisms. It is known that more than 20% of the human genes code for enzymes, responsible for basic cellular metabolism [1]. However, only a portion of the enzymes in the human genome have been characterized so far. Traditionally the study of enzymes or functional proteins is carried out on individual proteins recombinantly produced or purified from the original host, and thereby limited to only a few proteins at a time. This restricts the capability to systematically understand the many and potentially multi-functional roles of these proteins in the cell. In the past decade, the development of analytical technologies in genomics and proteomics have made it possible to systematically study gene regulation, protein expression and post-translational modifications on a large scale. This has advanced our understanding of complex biological systems tremendously. However, there remains a significant knowledge gap in our understanding and annotation of protein functions at a proteome level.

From a system biology and drug targeting perspective, it will be valuable if the functions of a family of enzymes or functional proteins such as proteases, protein phosphatases or kinases can be analyzed from a proteome simultaneously. For example, in the development of protein kinase inhibitors for cancer or other diseases, it will be valuable to determine if the drug candidate inhibits not just the target of interest but whether other protein kinases are impacted causing possible side effects. While protein kinase panels have been developed for inhibitor screening, these protein kinases are expressed in different host cells, such as *Escherichia. coli* or yeast, and the levels and status of the protein kinases in the panel may not reflect the actual status in wild-type cells, or cells associated with diseases like cancer. Another area that systematic analysis of enzyme function will bring value is cell metabolism because hundreds of metabolic enzymes are involved in metabolism. Recently, cancer metabolism has become a focused area for anti-cancer drug development [2]. Studies have shown an increase in the activity of the glycolytic enzymes in various type of tumors and cancer cell lines [3,4,5,6]. The systematic analysis of glycolytic enzymes in cancer cells could reveal more insights into cell metabolism and potential drug targets [2,7,8,9,10,11,12,13,14].

Nevertheless, a systematical analysis of protein functions from a complex proteome is still challenging. Recently several studies have reported various approaches to study protein function systematically. For example, a structure-based approach was taken to profile various enzyme families [15,16]. In this approach, chemically synthesized probes were used to study enzyme families such as serine hydrolases, cysteine proteases, and tyrosine phosphatases. These approaches included the use of a complex naturally derived proteome, and the proteins with high affinity towards the probes were isolated and further identified with LC-MS. Such approaches however rely on covalent modification to an exceedingly well-characterized structural feature. Notwithstanding these innovations, there remains need to systematically monitor the kinetics of protein function from complex proteomes in real time, and which can be linked to LC-MS identifications. Such methods will support the annotation of alternative functions within the same gene sequence and similar functions within different sequences, now vastly under-represented in proteomic annotations. New advantaged workflows that can support such systematic analyses for diverse enzyme families are described.

The methods described here combine bead-based enrichment towards selected sub-proteomes, followed by a modified 2-dimensional gel separation and electro-elution process called PEP. The PEP technology uses a modified Two-dimensional Gel Electrophoresis (2DE) to separate the proteome, without substantially compromising function [17]. The 2DE resolved proteins are then electro-eluted from the PEP plate into microwell plates and further refolded to regain enzymatic activity. These enzyme activities can then be measured systematically from hundreds to thousands of fractions depending on the complexity of the proteome and degree of resolution necessary for the profile and analysis.

## 2. Materials and Methods

### 2.1. Materials

All the chemicals were purchased from Sigma-Aldrich (St. Louis, MO, USA). Isoelectric Focusing (IEF) unit capable of running IEF at different lengths is from Bio-Rad (PROTEAN IEF Cell, Hercules, CA, USA). Spectrophotometer Plate Reader capable of reading 384-well plates with a wide wavelength selection and fluorescence reading is the SPECTRAMax Plus from Molecular Devices (Sunnydale, CA, USA).

### 2.2. Beef Liver Protein Extract Preparation

Frozen beef liver tissue was purchased from the local supermarket. Five grams of frozen beef liver tissue was chopped up with a razor blade and homogenized in 2.5 volumes of phosphate-buffered saline (PBS) using a disposable plastic homogenizer. After spinning at 14,000 g for 15 min, the supernatant was analyzed for protein concentration with BCA and frozen at −20 °C in small aliquots for single use.

### 2.3. AlbuVoid™ Treatment for Low Abundance Serum Protein Enrichment

Two hundred milligrams of AlbuVoid™ beads were used to process 0.8 mL of human serum (containing about 40 mg total serum protein), and separated with AlbuVoid™ according to the manufacturer’s instruction (Biotech Support Group, Monmouth Junction, NJ, USA) with one modification. In lieu of the supplied kit elution buffer, the enriched low abundance serum proteins were eluted with 0.8 mL elution solution containing 8 M urea, 2% CHAPS in 25 mM phosphate buffer, pH 8.0. The protein concentration was determined by BCA before 2-D gel electrophoresis.

### 2.4. KinaSorb™ Enrichment of Kinase and ATP Binding Proteins

KinaSorb™ is supplied in kit format including the activated beads, ATP for immobilization and Cleanascite™ suspension for phospho-lipid removal. The KinaSorb™ beads are supplied as a liquid suspension; 0.1 mL of KinaSorb™ was to treat 0.4 mL of human serum, according to manufacturer’s instruction (Biotech Support Group, Monmouth Junction, NJ, USA). Similarly, 0.1 mL of KinaSorb™ was used to treat 0.2 mL of beef liver (50 mg/mL). The enriched kinase (and other ATP binding) proteins were eluted with 0.8 mL elution solution containing 8 M urea, 2% CHAPS in 25 mM phosphate buffer, pH 8.0. The protein concentration was determined by BCA before 2-D gel electrophoresis.

### 2.5. Isoelectric Focusing (IEF) and 2-D Gel Electrophoresis

To prepare for the IEF separation, Bio-Lyte Ampholyte (Bio-Rad #1631112) were added to the eluates described above for a final concentration of 0.5%. These were then rehydrated with 0.4 mL sample solution with nonlinear pH 3–10 11 cm IPG strip (Bio-Rad ReadyStrip #1632016) overnight with a total loading of 1 mg protein/gel. The proteins were separated using the following setting: 0–7000 voltage gradient for 4 h, hold at 7000 voltages overnight until running termination, the IEF being run at room temperature. After IEF, the IPG strips were taken off the running unit, mineral oil from the IPG strip was absorbed with a paper towel and the IPG strip was transferred to a 12-lane refold tray (Bio-Rad #1654025). Four milliliters of refolding solution was added to each lane with the IPG strip and incubated for 10 min, this step will allow the urea to diffuse out of the IPG strip and also the refolding of the protein in the IPG strip, this was followed by incubation with electrophoresis transfer buffer (Tris-glycine with 0.1% SDS), this step will allow the further diffusion of urea from the IPG strip and most importantly the binding of SDS to the protein so that all the proteins become negatively charged.

For protein refolding, a protein refolding solution to mimic the cell cytoplasm was used. This solution contains the following component amounts per liter of the final volume: anhydrous CaCl_2_, 5–200 mg; anhydrous MgCl_2_, 15–50 mg; anhydrous MgSO_4_, 20–80 mg; FeSO_4.7H.sub.2O_, 0.05–0.50 mg; Fe(NO_3_)_3.9H.sub.2O_, 0.01–0.08 mg; ZnSO_4.7H.sub.2O_, 0.40–1.20 mg; ferric ammonium citrate, 0.04–200 mg; KCl, 280–500 mg; NaCl, 5000–7500 mg; d-Glucose, 500–8000 mg; sodium pyruvate, 0.0–1000 mg; sodium hypoxanthine, 0.0–20.0 mg; glycine, 0.0–150 mg; l-alanine, 0.0–150 mg; l-arginine.HCl, 200–5000 mg; l-asparagine.H_2_O, 40–250 mg; l-aspartic acid, 20–1000 mg; l-cysteine.HCl H_2_O, 25.0–250 mg; l-cystine.2HCl, 15–150 mg; l-glutamic acid, 0–1000 mg; l-histidine.HCl.H_2_O, 100–500 mg; l-isoleucine, 50–1000 mg; l-leucine, 50–1000 mg; l-lysine.HCl, 100–1000 mg; l-methionine, 50–500 mg; l-phenylalanine, 25–1000 mg; l-proline, 0–1000 mg; l-serine, 50–500 mg; l-taurine, 0–1000 mg; l-threonine, 50–600 mg; l-tryptophan, 2–500 mg; l-tyrosine.2Na.2H_2_O, 25–250 mg; l-valine, 100–1000 mg.

After protein refolding, the IPG strip was laid down to a precast 2-D gel (Bio-Rad 10%–20% Criterion Gel #3450107) with the acidic end of the IPG on the left side of the 2-D gel when facing the gel apparatus. The gel was operated at 80 volts for 15 min. followed by running at 120 volts until the dye front of the gel is 0.5 cm from the bottom edge of the gel.

### 2.6. Electroelution and Protein Recovery from the PEP Plate

After second dimension gel electrophoresis, the gel was taken out from the cassette, and laid on top of the PEP plate, which was filled with elution solution, containing a large molecule enzyme stabilizer. The proteins were transferred from the gel to the PEP plate for 60 min at 20 volts using a Semi-Blot apparatus from Bio-Rad (#1703940.). After protein transfer, the gel was carefully lifted from the PEP plate, and a multi-channel pipette transferred the eluted proteins from the PEP plate to a master plate, containing 50 μL PBS in each well. About 40–45 μL of solution could be transferred to the Master Plate for a total volume of 90–95 μL in each well. In this analysis, 25 μL solution was taken from each well in the Master Plate and transferred to an enzyme assay plate to perform the enzyme assay.

### 2.7. Hexokinase Activity Assay

Hexokinase activity can be monitored by a cascade reaction as follows:

Hexokinase
Substrates added {d-Glucose + ATP} → Products {d-Glucose 6-Phosphate + ADP}

G-6-PDH d-Glucose 6-Phosphate + ß-NADP → 6-Phospho-d-Gluconate + ß-NADPH

In the final assay solution, glucose was at 216 mM; MgCl_2_ at 7.8 mM, ATP at 0.74 mM and NADP at 1.1 mM. 25 μL of this enzyme assay solution was mixed with 25 μL of sample from the Master Plate (described above) and the enzyme activity was monitored by the 340 nm absorbance from the reduction of NADP to NADPH. The readings at 0, 1 h, 3 h and overnight measurements were recorded. However, in lieu of purified G-6-PDH, 0.25 mg/mL beef liver protein was used as the source of Glucose-6-Phosphate Dehydrogenase (G-6-PDH). The assay thus reports the additive contributions of the endogenous Hexokinase activity present in the beef liver extract, and any exogenous activity from the presence of test sample protein in the PEP plate, which may influence the reduction of NAD or NADP (the reporting signal). In light of the ambiguities that may arise from such a reporting system, the primary goal of this investigation was to generate sufficient signal intensities and activity features which could be monitored and compared across samples types within an “omics” context. Therefore, this broader spectrum assay was chosen over a more narrow spectrum substrate to product turnover one, as this broader spectrum assay could potentially detect the activities of downstream glycolytic and other cross-regulating proteins from the test samples.

## 3. Protein Kinase Activity Assay

Protein kinase activity was measured using the ADP-Glo kit from Promega (Catalog Number: V9102). A volume of 22.5 µL each, of the PEP plate sample and protein kinase substrate mixture, were added together. Reaction was carried out at room temperature for 2 h. Ten microliters each of reaction mixture and ADP-Glo and 20 µL of Kinase Detection Reagent for the final detection according to the manufacturers’ directions (Promega, Madison, WI, USA). Luminescence was monitored at 8 reads/well.

## 4. Alkaline Phosphatase Activity

A pNPP Microwell substrate system from KPL (KPL Ltd., Gaithersburg, MD, USA) was used to measure Alkaline Phosphatase. Briefly, 22.5 µL each of the sample from PEP plate and substrate was mixed and incubated at room temperature. The absorbance was monitored spectrophotometrically at 405 nm.

## 5. Protease Activity

FITC-labeled casein was used as general protease substrate at 0.5 mg/mL final concentration.

Twenty five microliters each of the PEP plate sample and substrate were incubated at room temperature overnight in the dark, after protease digestion, the casein was precipitated with 10% TCA (trichloroacetic acid) and the supernatant was neutralized with Tris base and used for the fluorescence measurement.

Proteases Assay:
Casein (substrate)-FITC → Hydrolyzed Casein + FITC

### Enzyme Activity Display

Two Microsoft Excel formats were used to display the enzyme activities. One is to use the 3-D column display and the other is the heat map.

## 6. Results and Discussion

**Figure 1:**
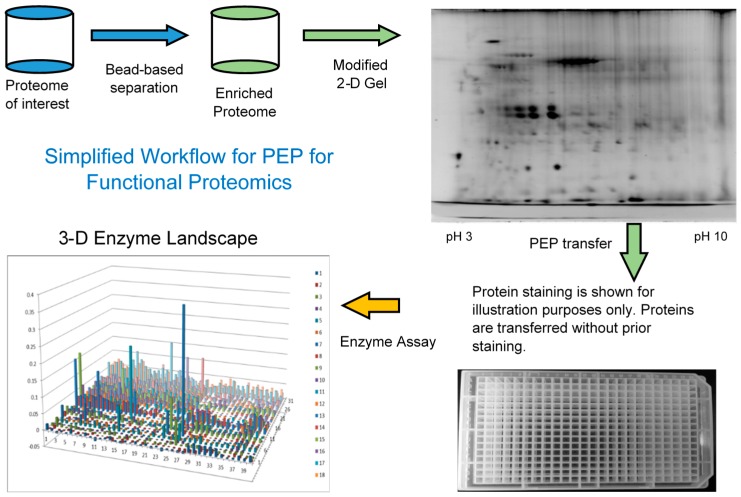
Simplified workflow of the combined enrichment and the PEP technology.

Since most of the functional proteins or enzymes exist at relatively low levels and there is a limited loading capacity on the 2-DE gel, it can be beneficial to first enrich the low abundance proteins before 2-DE and PEP analysis, Figure 1. AlbuVoid™ and KinaSorb™ are both shown to effectively enrich low abundance proteins while depleting the high abundance proteins, albumin in the case of serum. It is important to note that the proteins obtained after treatment with both of these products, are functional (not denatured) and suitable for the modified 2DE and final enzyme activities to be analyzed.

The selection of the activity to be monitored is an important choice in the functional proteomic investigation. Our methods require protein refolding, a phenomena usually obtained for structurally simple enzymes. Non-denaturing modifications to the first dimension separation may be necessary for complex, multi-subunit enzymes like Proteasome. That is an area for future investigation. While such refolding challenges are acknowledged, the functional analysis does not need a complete refolding back to wild-type activities, for the described methods to be productive. Ultimately, it is the constellation of enzyme activity features that drives the investigation. Because many enzyme assays are very sensitive—with detection as low as picogram amounts of protein, even just a residual amount of activity will allow sufficient feature development to compare and contrast biological samples.

Thus, subject to the limits of refolding, and depending upon the goals of the investigation, the PEP technology allows for an open and exceedingly diverse selection of enzyme substrates. It can be adapted to most common quantitative instruments to monitor either spectrophotometric, fluorimetric, or chemiluminescent reporting signals generated by product formation. It is important to note that the choice of substrate will drive the application and goals of the investigation: Broad spectrum substrates will produce more profile features, and may be best suitable for pure data driven biomarker discovery applications. Narrow spectrum substrates produce less features, and this may prove advantageous when cataloging or annotating subset proteomes with similar functional attributes, or any hypothesis driven biomarker discovery project.

In the current study, we considered several substrates that followed this narrow to broad spectrum line of reasoning. First, a specific assay for hexokinase activity was measured, with only two substrates (glucose and ATP) introduced at optimized conditions [18]. Hexokinase activity was selected for several reasons. Foremost is that the products produced from Hexokinase activity are the first within the glycolytic pathway, a pathway often implicated in cancer development [18,19]. Another reason is that a large number of functional proteins within and cross-regulating with the glycolytic pathway could potentially be monitored by a broad spectrum assay as the one employed, which already contains endogenous Hexokinase activity. The introduction of exogenous protein(s) from the PEP samples could potentially supersede any rate-limiting protein function and enhance the hexokinase activity. As such, this assay may also detect the effect of proteins from other pathways that cross-interact with the glycolytic pathway.

Likewise, to illustrate the versatility of the platform, we report on protein kinase and protease activity, on a complex substrate—casein, along with alkaline phosphatase activity.

Because of the extensive post-translational modification of the proteome, proteins with the same abundance can and often have very different activities. Enzymes can vary with respect to substrate specificities and kinetics. For example, many protein kinases will be activated by phosphorylation promoting a functional modulation even when the protein abundance may be the same. Splice variants, sub-unit makeup and non-covalent regulating factors, can also influence enzyme activity. Thus, while quantitative efforts in proteomics are promising, a critical assumption is that function is linearly proportional to protein abundance. However, conformational variability affects functional activity in a non-linear fashion.

The discovery of intrinsically disordered proteins (IDPs) and hybrid proteins consisting of ordered domains and intrinsically disordered protein regions (IDPRs) challenged the protein structure paradigm stating that a protein must have a defined 3D-structure, based solely on amino acid sequence in order to perform a function [20]. Intrinsically disordered sequence cycles through a continuum of conformations. Mutations, post-translational modification, and non-covalent binding factors all play a role in fine tuning the polypeptide sequence to final function [21]. Because of this phenomena, populations of proteins annotated to the same sequence nevertheless can display multiple functions within tissues and disease states. α-enolase serves as an example of one such “moonlighting protein”, one of a growing list of proteins that are recognized as identical gene products exhibiting multiple functions at distinct cellular sites [22]. Likewise, the same or similar function may be presented by multiple sequences, with subcellular control mechanisms regulating functional diversity. As a consequence, strictly abundance based biomarkers may lack the high dynamic range and greater specificity potentially provided by functional based biomarkers, to define the phenotype. Thus, Functional Proteomic techniques such as proposed here, support a top-down proteomic strategy starting with functional annotation of the structurally intact protein, and ending with sequence and structural annotation.

**Figure 2:**
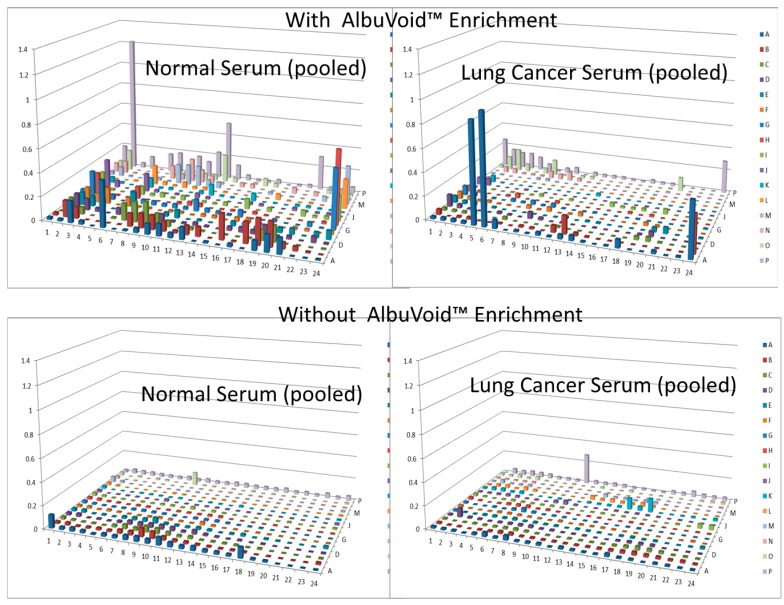
Hexokinase activity of normal and lung cancer patient serum, with and without AlbuVoid™ enrichment.

**Figure 3:**
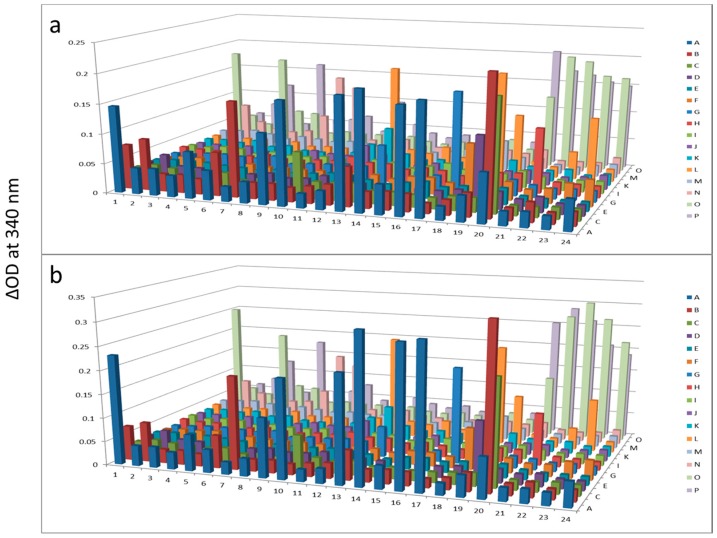
Hexokinase activity at 180 min (**a**) and 300 min (**b**) after KinaSorb™ treatment of human serum. The readings at 340 nm were from each of the 384-well microplate with a portion of the proteins recovered from the PEP plate.

**Figure 4:**
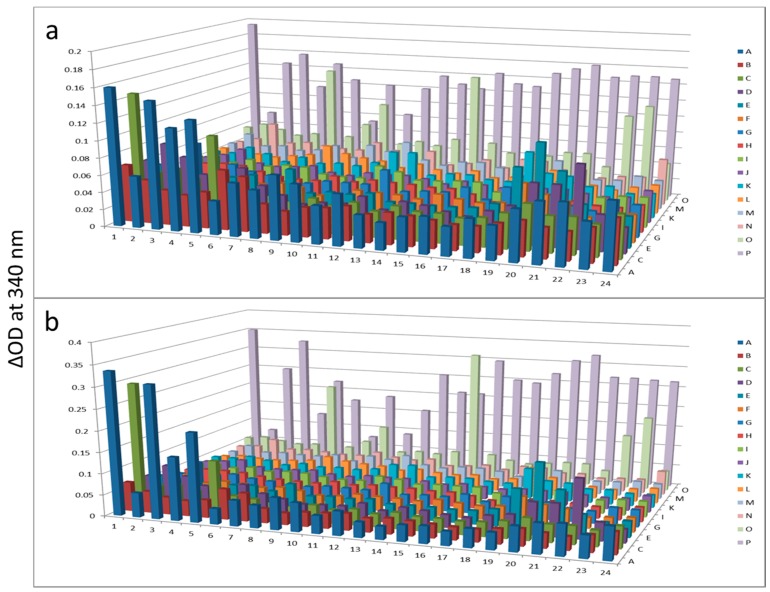
Hexokinase activity at 180 min (**a**) and 300 min (**b**) after KinaSorb™ treatment of beef liver protein. The readings at 340 nm were from each of the 384-well microplate with a portion of the proteins recovered from the PEP plate.

**Figure 5:**
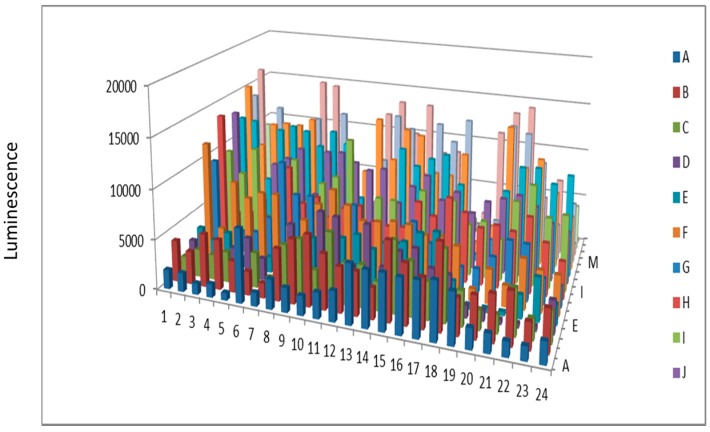
Protein kinase from beef liver after KinaSorb™ enrichment.

**Figure 6:**
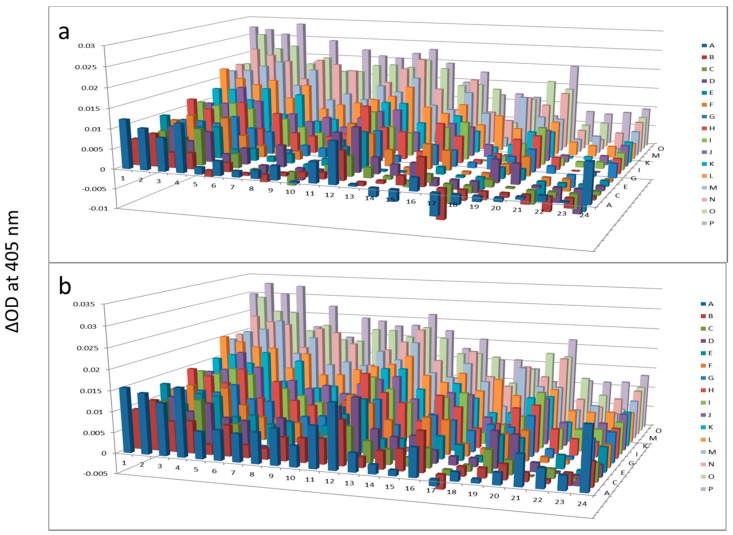
Alkaline phosphatase after AlbuVoid™ treatment of human serum at 60 min (**a**) and 120 min (**b**). The readings at 405 nm were from each of the 384-well microplate with a portion of the proteins recovered from the PEP plate.

**Figure 7:**
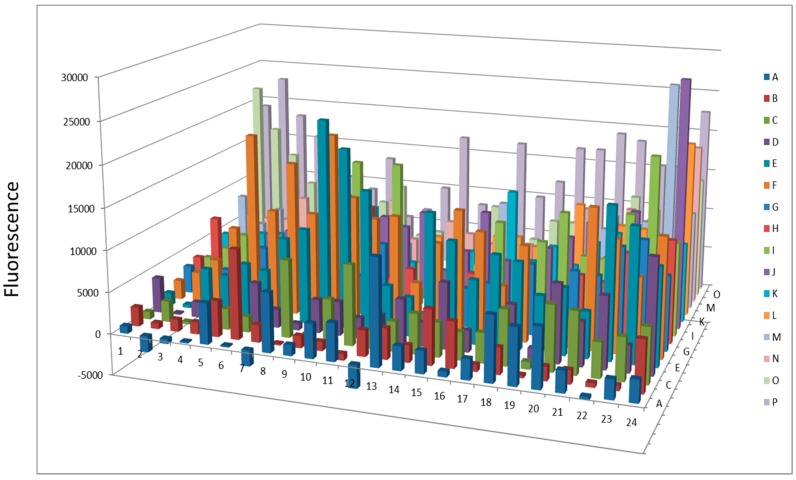
Protease activity overnight after AlbuVoid™ treatment of human serum.

In this short case study, we demonstrate that enrichment of select sub-populations of proteins is beneficial to systematically analyze protein functions of a whole enzyme family from an entire proteome. In Figure 2, AlbuVoid™ was used to remove Albumin and enrich the low abundance proteome, noting that distinguishable features are presented from the lung cancer *vs.* the normal sera. In Figure 3, Figure 4 and Figure 5, KinaSorb™ was used to enrich for both a narrow spectrum substrate profile—Hexokinase activity, and a broad-spectrum protein kinase activity. The number of observable features was consistent with such narrow and broad-spectrum activities. In Figure 6 and Figure 7, AlbuVoid™ enrichment and PEP processing proved suitable for profiling the functional activities of Hexokinase, Protease and Alkaline Phosphatase. These enzyme feature profiles are indicative of the functional diversity that can be generated, annotated and compared within and between sample phenotypes.

As a proof-of-concept, two samples (the first one and the last one) collected from the lung cancer patient hexokinase analysis, among the 54 samples that showed significant difference between normal and cancer patient were subjected to Mass Spectrometry analysis. Both samples were identified as glyceraldehyde-3-phosphate dehydrogenase. This is not surprising as the hexokinase assay was set up in such a way that any enzymes downstream of hexokinase that can positively (by removing a product) or negatively (by product feedback inhibition) impact hexokinase turnover can potentially be detected in this assay. An extensive mass spectrometry analysis of samples identified in lung cancer biomarker studies and further biological validation will be presented in the near future.

As a result, such functional annotation will be complementary to that acquired through gene expression and protein abundance analyses. For drug development, the functional landscape of a proteome will likely improve compound specificity while minimizing promiscuous interference with other protein functions. This can be a confounding problem when post-translational modifications activate protein function, of which would not otherwise be observable if only protein abundance was monitored.

In addition, because of gene editing and post-translational modification, almost all functional proteins including enzymes have isoforms. Therefore, these technologies will be valuable tools in the analysis of the function of enzyme isoforms to further understand their biochemical functions, compartmentalization, sequence regulation, and potentially therapeutic modulation in different tissues and diseases.

This methods development study demonstrates the beneficial elements of combining bead-based enrichment products upfront to PEP, generating many detectable features within the derived functional profiles. These methods support LC-MS protein identification, and confirm a previous report demonstrating that the proteins isolated from the PEP process are sufficiently pure and can be identified by mass spectrometry [17]. Consequently, future studies with these combined methods will include protein identification and assignments as part of the study. Such gene identifications will supplement the functional activities to provide a comprehensive proteome characterization of the disease or tissue phenotype of interest. These new methods thus enhance the study of functional diversity now open to any proteome investigation, limited only by the availability of substrates and sensitive ways to monitor, *in vitro*, the conversion of substrates to products.
